# Effect of Fe and Cr on the Macro/Micro Tribological Behaviours of Copper-Based Composites

**DOI:** 10.3390/ma14123417

**Published:** 2021-06-20

**Authors:** Zhongyi Zhang, Haibin Zhou, Pingping Yao, Kunyang Fan, Yongqiang Liu, Lin Zhao, Yelong Xiao, Taimin Gong, Minwen Deng

**Affiliations:** 1 State Key Laboratory of Powder Metallurgy, Central South University, Changsha 410083, China; zhangzhongyi1979@126.com (Z.Z.); zhaolin@csu.edu.cn (L.Z.); xiaoyelong87@126.com (Y.X.); gongtaimin@csu.edu.cn (T.G.); 18874108043@163.com (M.D.); 2 School of Mechanical Engineering, Chengdu University, Chengdu 610106, China; fankunyang123@126.com; 3 Aerospace System Engineering Shanghai, Shanghai 200000, China; heru689@163.com

**Keywords:** abrasion component, interface, tribological performance, Cu-based composites

## Abstract

Fe and Cr are regarded as two of the most important friction components in Cu-based composites (Cu–BCs). In this study, the microstructural detection and micro- and macro-tribology evaluation of Cu–BCs containing Fe and Cr were performed. The results indicated that both Fe and Cr formed diffusion interfaces with the copper matrix. Because of the generation of a defect interface layer, the Cr/Cu interface exhibited a low bonding strength. Owing to the excellent binding interface between Fe and Cu, the high coefficient of friction (COF) of Fe, and the formation of a mechanical mixing layer promoted by Fe, the Cu–BCs containing Fe presented better friction performance under all braking energy per unit area (BEPUA) values. The main wear mechanism of Cu–BCs containing Fe and Cr changed from abrasion to delamination with an increase in BEPUA, and the delamination of Cu–BCs containing Fe was induced by breaks in the mechanical mixed layer (MML).

## 1. Introduction

Cu-based composites for brake applications are generally composed of three different components: matrix components, lubrication components, and friction components [1]. The matrix components control the basic physical and mechanical properties. Copper is widely used as a matrix component because of its good ductility, electrical conductivity, and thermal conductivity [2,3,4]. The lubrication components are used for smoothing the braking process and the friction components, which determine tribological performance, play an important role in Cu–BCs [5,6,7].

Fe and Cr are the most crucial friction components owing to their low solubility and high wettability with the copper matrix, which effectively increase the temperature resistance, resistance to plastic deformation, coefficient of friction, and wear resistance of Cu–BCs. Therefore, Cu–BCs containing the friction components mentioned above are utilized in high-energy braking systems for aerospace, high-speed trains, and heavy-duty engineering machinery applications [8,9].

To analyse the role of Fe and Cr for Cu–BCs in detail, several studies have been carried out. Peng et al. [10,11] determined that a high Fe content helps the formation of oxidative film, thereby improving the wear resistance and stability of the coefficient of friction (COF) of Cu–BCs during braking. Zhang et al. [12] reported the friction characteristics of Fe types in Cu–BCs, and the results showed that the performance of reduced Fe powder was better than that of carbonyl Fe powder and flake Fe powder thanks to the former’s ability to maintain a high and stable COF under high-energy braking. Zhong et al. [13] studied the effects of Fe content on the tribological performance of Cu–BCs, and the conclusions showed that an increasing Fe content contributed to friction torque stabilization, COF growth, and wear reduction. However, it was easy to produce a sharp drop in the wear performance of Cu–BCs with the addition of excess Fe. Shen [14] studied mechanical properties of Cu–Fe interfaces, and the results showed that Cu (111)/Fe (110)-NW and Cu (111)/Fe (110)-KS interfaces have higher strength, which increases the tensile strength of the materials. Wang [15] studied the relationship between the tensile property and the γ-Fe/Cu interface, and the conclusions showed that the Fe (110)/Cu (110) interface shows a higher strength metal bond, which showed that Fe accelerate nucleation and growth of Cu.

The functions of Cr are different from those of Fe. Cr is the hardest metal in nature; at the same time, Cr has a high melting point, strong corrosion resistance, and oxidation resistance. This type of friction component is utilized for raising hardness, improving electrical properties, and strengthening oxidation resistance of Cu–BCs [16,17]. Gao [18] studied the influence of Cr content on the friction and wear properties of Cu–BCs, and the results showed that COF gradually increased with Cr content in the range of 3–5 wt.%. However, Fang [19], who obtained opposite results, noted that an increase in Cr content reduced the wear of Cu–BCs, but also decreased COF, especially under conditions of 15–20 wt.% Cr content. Fu et al. [20] argued that Cr not only contributed to an increase in COF, but also enhanced wear resistance. Chen [21] studied that the relationship between the tensile strength and Cu/Cr interface of Cu-Cr-Zr alloy, and the results showed that a semi-coherent interface was formed of Cu/Cr, which increased the strength of Cu-Cr-Zr alloy and suppressed the formation of dislocations.

In summary, the current research mainly focuses on the influence of Cr and Fe on mechanical properties and the macro friction and wear performance of Cu–BCs, whereas the intrinsic micro tribology behaviours of Fe, Cr, and their interface with matrix are still not fully understood. In addition, influences of Fe and Cr on high energy braking performance of Cu–BCs are not clear as well. Therefore, it is necessary to carry out a comprehensive and detailed study on micro–macro perspectives on the action mechanisms of Fe and Cr in Cu–BCs. This study aims to systematically investigate the differences between Fe and Cr regarding their interface characteristics with the copper matrix, as well as their micro and macro tribology properties. The purpose of this research is to clarify in detail the influence of friction components Fe and Cr on tribology behaviour of Cu–BCs, specially under high braking energy conditions. The findings of this work will contribute to the development of high performance braking materials.

## 2. Experimental Section

### 2.1. Materials

Table 1 lists the compositions of Cu–BCs prepared by the powder metallurgy method. The raw powders included reduced Fe and Cr with 99.85% purity manufactured by Jinjiang Powder Metallurgy Factory (Quanzhou, China), electrolytic Cu powder (99.98%) from the Huahao company (Chongqing, China), and KS150-600SP graphite powder of 97% purity (Imerys Graphite & Carbon Company, Paris, France); these powders were used as the raw materials. The characteristic parameters of these powders are listed in Table 2.

**Figure 1:**
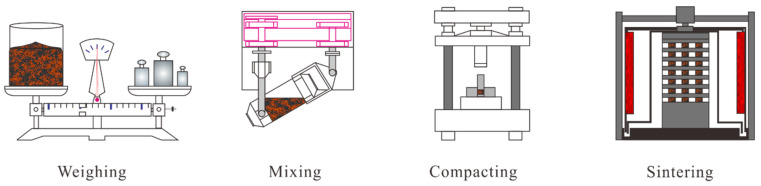
Preparation process for Cu–BCs.

The production process for Cu–BCs is presented in Figure 1. The preparation process of the experimental samples was as follows: (1) weighing raw powders according to the formula in Table 1; (2) mixing raw powders in a roller mixer for 6 h; (3) pressing the mixed powders in moulds under a pressure of 400 MPa; and (4) sintering green compacts using home-made pressure sintering furnace with a pressure of 2.5 MPa at 970 °C for 3 h. During the sintering process, hydrogen was selected as a protective atmosphere to prevent oxidation of metal at a high temperature.

In order to ensure that Cu-Fe and Cu-Cr have roughly the same relative density, the ratio of *ρ/ρ_th_* needs to be calculated. It is calculated as follows:

(1) $$\rho_{th}=\frac{\rho_{1}V_{1}+\rho_{2}V_{2}}{V_{1}+V_{2}}$$

where *ρ*_1_, *ρ*_2_—component density; *V*_1_, *V*_2_—component volume.

(2) $$\rho_{r}=\frac{\rho }{\rho_{th}}$$

where *ρ_r_*—relative density; *ρ*—material measured density; *ρ_th_*—theoretical density.

After sintering is complete, densities (*ρ*) of the Cu–BCs were measured according to the drainage method. *ρ_th_* is calculated according to Equation (1). The relative density (*ρ/ρ_th_*) and density (*ρ*) as shown in Table 3.

### 2.2. Experimental Methods

Microstructural characterizations of the experimental samples and bonding interfaces were surveyed using a Leica-Q550 metallurgical microscope (Leica Camera AG, Wetzlar, Germany) and a scanning electron microscope (FEI Quanta 250 FEG SEM, FEI company, Hillsboro, OL, USA) equipped to examine the energy dispersion spectrum.

The micro tribological properties of the Fe and Cr phases and interface strength were measured by a micro friction tester (manufactured by CSM instrument, Acacias, Switzerland). The samples were machined into cylinders about 25 mm in diameter and then polished until surface roughness values (Ra) of 1.2 were attained before testing.

The micro-friction test was carried out in three stages: pre-scan of the indenter, friction test, and data calculation. Because the experimental procedures were described in our previous work, the detailed test procedures will not be elaborated on in detail here [22]. In this paper, we used the same test method and simplified the test parameters. Table 4 lists the key parameters for the micro-friction tests. The COF of tests was measured as

(3) $$\mu =\frac{F_{t}}{F_{n}}$$

where *F_t_* is the tangential force and *F_n_* is the normal force. The detailed test parameters are shown in Table 4.

**Figure 2:**
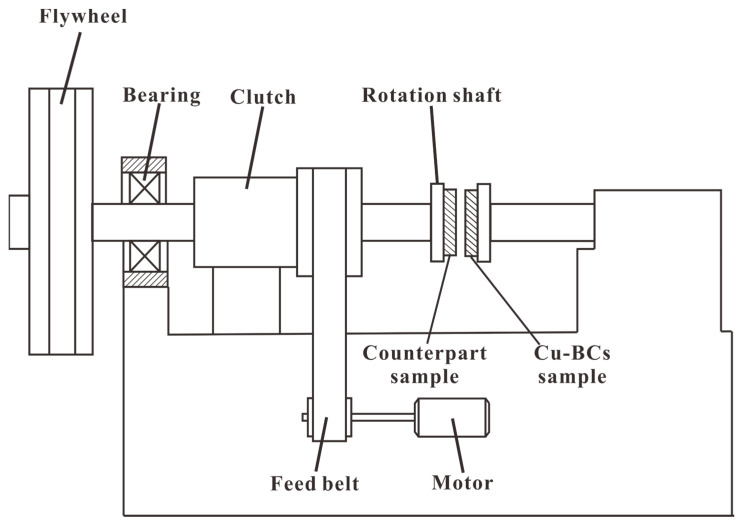
Structure chart of the MM-3000 friction tester.

The influences of Fe and Cr on the macro tribological properties of Cu–BCs under different BEPUA were evaluated by a braking tester (MM-3000, Shuntong electromechanical technology research institution, Xi’an, China), as shown in Figure 2. Next, 30CrMnSiV steel alloys containing 0.27–0.34% C, 0.8–1.1% Mn, 0.8–1.1% Cr, 0.3–0.4% V, 0.9–1.2% Si, *p* ≤ 0.0035%, and S ≤ 0.03% were adopted as counterparts, which shows a Rockwell hardness of 39–42. All samples and counterparts were machined into rings with an inner diameter of 53 mm and an outer diameter of 75 mm. These rings were then polished until surface roughness values (Ra) of 1.2 were attained before testing. The macro friction parameters are given in Table 5. According to the test parameters, the test conditions can be divided into low BEPUA (3000 rpm, 8.47 J/mm^2^), medium BEPUA (5000 rpm, 23.50 J/mm^2^), and high BEPUA (46.10 J/mm^2^).

## 3. Results and Discussion

### 3.1. Morphology, Microstructure, and Interface Characteristics

**Figure 3:**
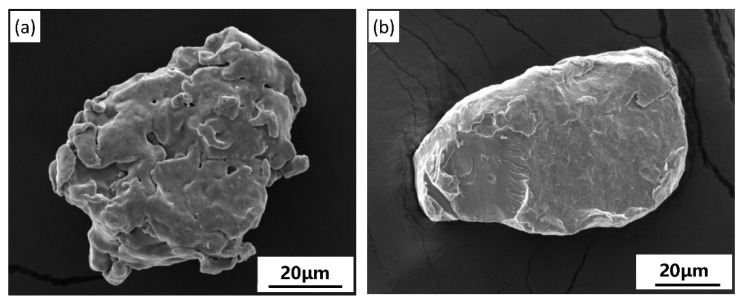
Typical morphology of the Fe and Cr particles: (**a**) Fe particle and (**b**) Cr particle.

The shape of Fe and Cr particles was generally similar, but the morphologies were different. The Fe powder had a typical spongy and spherical-like structure. However, compared with the Fe powder, the Cr powder possessed a denser and smoother particle morphology, as shown in Figure 3b, which was mainly caused by the small volume shrinkage during the reduction of Cr.

**Figure 4:**
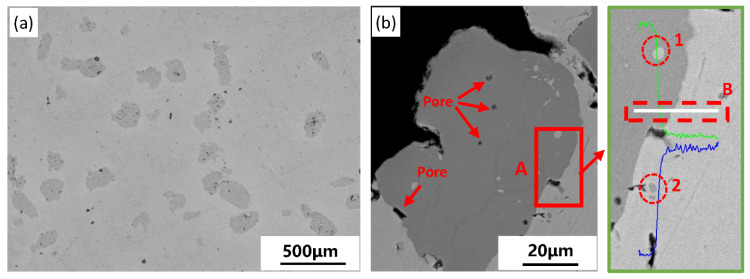
Microstructure of the Cu–Fe sample (**a**) and the characteristics of the Fe/Cu interface (**b**).

**Figure 5:**
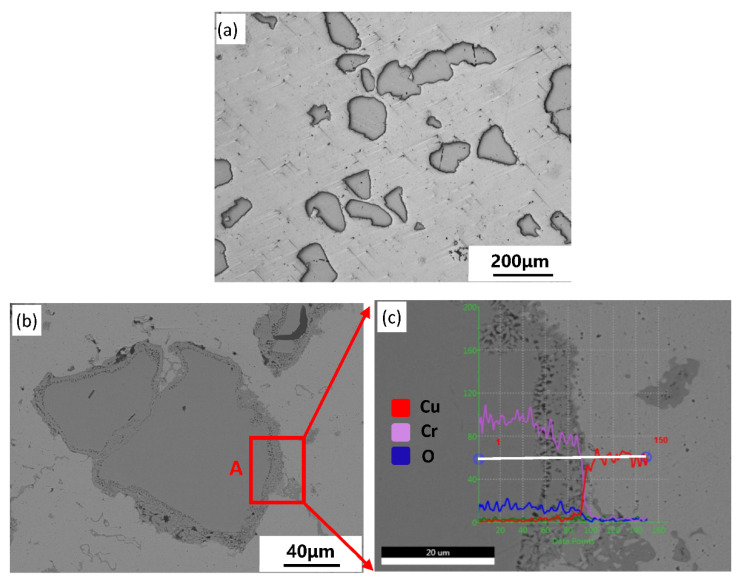
Microstructure of the Cu–Cr sample and interface characteristics. (**a**) Microstructure of the Cu–Cr sample (A2); (**b**) enlarged image of the Cr friction component; (**c**) characteristics of the Cr/Cu interface.

Micrographs of the Cu–Fe composite are shown in Figure 4a, which indicates that the grey Fe particles were uniformly distributed in the matrix. Figure 5b provides further illustrations of the Fe/Cu interface. Notably, a tightly bonded interface formed between the Fe phase and Cu matrix because of the excellent wettability between them. Nevertheless, a small number of microvoids with high curvature were present on the interface owing to vacancy aggregation, which is caused by the Kirkendall effect.

The interface morphology of the Fe/Cu interface is shown in detail in Figure 4b. According to the EDS scanning results of line B, the content of Cu and Fe changed rapidly in the interface, indicating no formation of an obvious diffusion layer in this area. However, the EDS analyses of point 1 (light Cu-rich phase inside Fe) and point 2 (dark Fe-rich phase inside Cu matrix), as shown in Table 6, indicated that mutual diffusion between Fe and Cu occurred during the sintering process. The generation of Cu-rich phases and Fe-rich phases was caused by a decrease in solid solubility between the two elements after the cooling process based on the Fe–Cu phase diagram [23].

Figure 5 shows the interface characteristics and the microstructure of the Cu–Cr composite. There was an obvious interface layer generated in the Cr/Cu interface, showing a completely different structure from the Fe/Cu interface. To determine the detailed features of the Cr/Cu interface, we employed micrography at high magnification, as shown in Figure 5c. The interface layer showed a pore-filled structure with 7–10 μm thickness. The EDS results of line 3, on which the Cr and Cu composition gradients changed, indicate that the mutual diffusion between Cr and Cu was stronger than that between Fe and Cu.

According to the analysis mentioned above, both Fe and Cr formed a diffusion bonding interface with the matrix. However, there were noteworthy differences between the two kinds of interfaces in morphology and structure owing to differences in the diffusion rate. For Cu–Fe composites, with an increase in the sintering temperature, the crystals of Fe changed from BCC structures to FCC structures, leading to an improvement in the solid solubility between γ-Fe and Cu. However, the extremely low inter-diffusion rate between the two phases resulted in the formation of a diffusion bonding interface without the interface layer. For Cu–Cr composites, the formation of interface morphology may lead by a combination of multiple mechanisms. The first influence factor is the inequality diffusion between Cu and Cr. When the temperature was raised to 970 °C, the solid solution of Cu within the Cr (bcc) phase was less than 0.25 at.%, but the maximum solid solution of Cr within the Cu (fcc) phase was close to 0.9 at.% [24]. In addition, according to the diffusion constants and activation energies of diffusion at sintering temperature, the bulk diffusivity of Cr towards Cu (*D_Cr–Cu_*: 2.32 × 10^−13^) was two orders of magnitude higher (*D_Cu–Cr_*: 6.93 × 10^−15^) than that of Cu towards Cr. Therefore, the diffusion fluxes of Cr within Cu were considerably higher than those of Cu in Cr. However, owing to a low mutual solubility between Cr and Cu, the effect of Kirkendall in interface morphology is limited. In this condition, the generation of pores in Cr/Cu interface may also be attributed to the precipitation of supersaturated hydrogen from metals, or the producing of undischarged water vapor formed by the reduction of oxide of Cr particles during the sintering process. Eventually, a pore-filled diffusion layer was formed in the Cr/Cu interface.

### 3.2. Microstructure and Mechanical Properties of Cu–BCs

**Figure 6:**
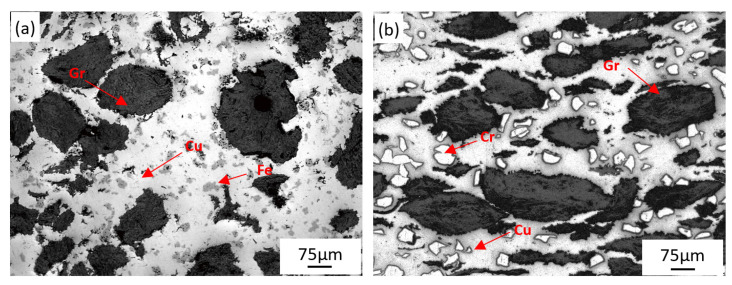
Microstructure of Cu–BCs: (**a**) Cu-Fe-Gr and (**b**) Cu-Cr-Gr.

Figure 6 shows the microstructure of Cu-Fe-Gr and Cu-Cr-Gr materials. Fe, Cr, and Gr were evenly distributed in the Cu matrix. Among them, the black bulky phases were granular graphite, which were used to reduce the vibration and stabilization braking. Light dark irregular phases in Figure 6a and white granular phases in Figure 6b were Fe and Cr respectively, which were used for improving tribology performance and reducing wear resistance.

**Figure 7:**
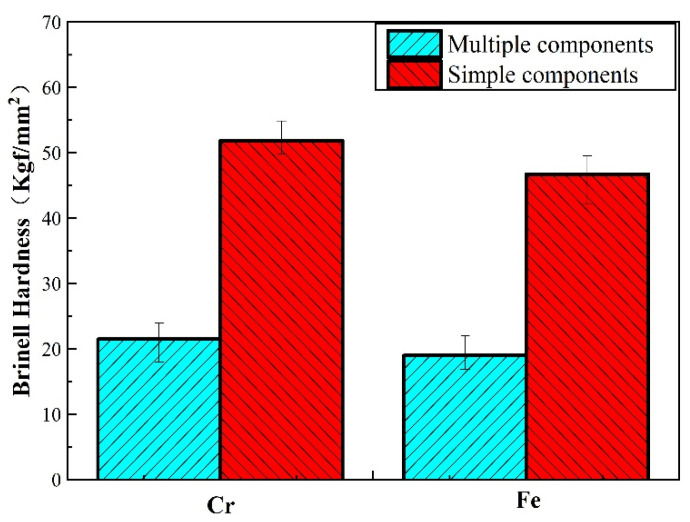
Brinell hardness of Cu–BCs.

The Brinell hardness of Cu–BCs is presented in Figure 7. Fe and Cr phases had a hardness of HV_0.1_ 109 N/μm_2_ and HV_0.1_ 248 N/μm^2^, respectively. Owing to the diffusion bonding interface formed between the friction component and matrix, the addition of a friction component can effectively improve the hardness of composites by means of particle strengthening. Between them, because of higher hardness, Cr showed a better particle strengthening effect than Fe, though its interface strength is relatively lower. It can also be noticed that, owing to the low strength of the lubrication component, the addition of graphite led to an obvious decrease in the hardness of Cu–BCs.

### 3.3. Micro-Friction Tests

During the braking process, macro friction can be regarded as the accumulation of micro friction. Therefore, an analysis of the micro friction performance of Fe and Cr components and their interface was needed to better understand the macro-tribological behaviours of the friction materials.

#### Tribological Properties of the Fe and Cr Phase and Its Interface

**Figure 8:**
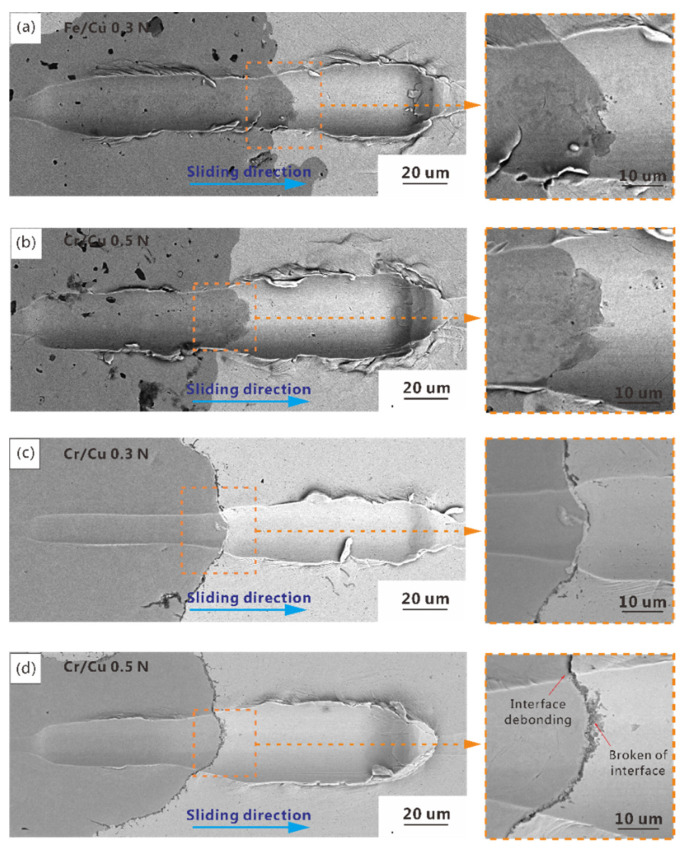
Scratch grooves of the Fe/Cu interface and Cr/Cu interface with different forces: (**a**) 0.3 N Fe/Cu interface; (**b**) 0.5 N Fe/Cu interface; (**c**) 0.3 N Cr/Cu interface; and (**d**) 0.5 N Cr/Cu interface.

Figure 8 presents the scratch grooves across the Fe/Cu and Cr/Cu interfaces under different levels of normal force. The widths of the grooves noticeably increased when the indenter slid from the friction component to the Cu matrix. However, the width increase of the Fe–Cu composite was relatively small compared with that of the Cr–Cu composite. When the normal force increased gradually, there was also significant growth in the widths of the grooves, especially for the copper matrix under normal force of 0.5 N. The morphology of the groove in the interface bonding area is illustrated in detail on the right side of Figure 8. For Fe–Cu composites, distinct interface deformation occurred at the Fe/Cu interface along the sliding direction under all test conditions. Notably, the Fe/Cu interface still maintained good interface bonding after the scratch process. Compared with the Fe/Cu interface, although the deformation degree of the Cr/Cu interface was smaller, partially broken and local interfacial debonding began to occur when the normal force reached 0.5 N.

**Figure 9:**
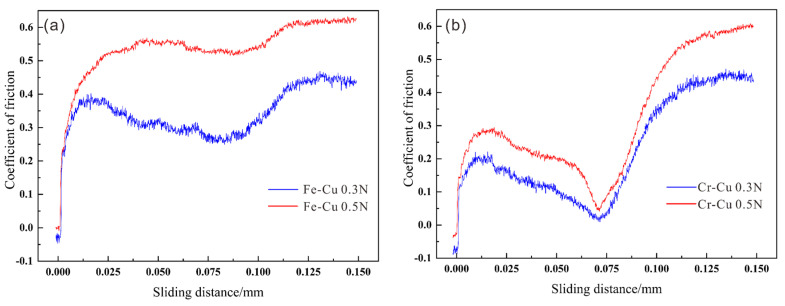
Curves of the coefficient of friction plotted versus the sliding distance in the Cu/Fe and Cr/Cu interface regions under different applied loads: (**a**) Fe/Cu interface regions and (**b**) Cu/Cr interface regions.

**Figure 10:**
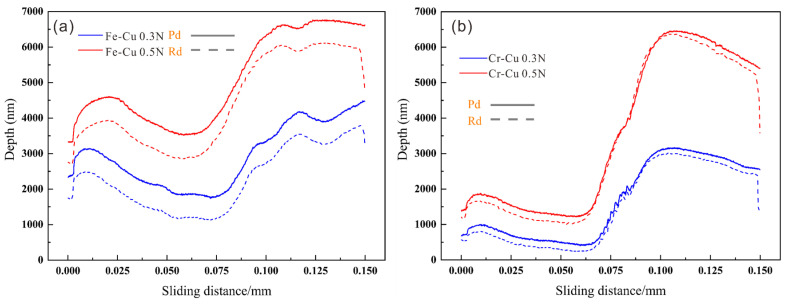
Curves for the *h_rd_*, *h_pd_* of friction plotted against the sliding distance in the Fe/Cu and Cr/Cu interface regions under different forces: (**a**) Cu/Fe interface regions and (**b**) Cu/Cr interface regions.

Figure 9 shows the COF curves with sliding distance under different levels of normal force. The value of COF was positively correlated with normal force. However, the different interface types showed completely different change tendencies in COF. For Fe–Cu composites, the COF increased slowly when the indenter slid across the Fe/Cu interface, especially under a pressure of 0.5 N. For Cr–Cu composites, the COF first showed a decreasing tendency and then an increasing tendency. When the normal force reached 0.5 N, the COF displayed a sharp decrease at the Cr/Fe interface. Figure 10 exhibits the penetration depth (Pd) and Rd (residual depth) curves with sliding distance under different normal forces. Notably, the Pd and Rd curves show similar change patterns to the COF curves. However, the changes in the Pd and Rd curves showed a slight difference in the interface area.

**Figure 11:**
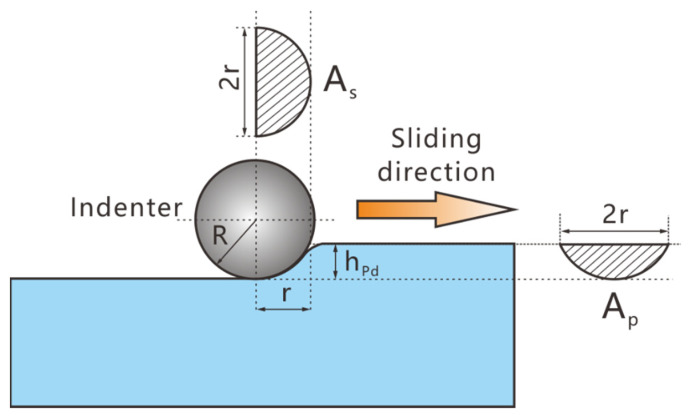
The schematic of indenter scratching on Cu–BCs.

Figure 11 displays a schematic of the micro-friction tests. According to the adhesive friction theory, the COF can be obtained as [25]

(4) $$\mu =\mu_{p}+\mu_{a}$$

where *μ_p_* is the COF of ploughing and *μ_a_* is the COF of adhesion. Owing to the poor adhesive tendency between the indenter and Cu–BCs, *μ_p_* plays a leading role during the sliding process. Moreover, for *μ_p_*, the values of Pd and Rd are close to each other, indicating that plastic deformation is the main deformation mechanism during the sliding process (elastic recovery is limited). Thus, the COF of ploughing can be given as [26,27,28]

(5) $$\mu_{p}=\frac{f_{p}}{N}=\frac{A_{p}H_{p}}{A_{s}H_{s}}$$

where *A_p_* and *A_s_* are the projection areas of the contact region along the horizontal and vertical directions, respectively, as shown in Figure 11. *H_p_* and *H_s_* are the plough hardness and scratch hardness of the friction components or Cu matrix, which are defined as the deformation resistance per unit projected area along the horizontal and vertical direction, respectively. Thanks to the homogeneity of the friction components and the matrix, *H_p_* and *H_s_* can be regarded as identical, which means that the COF of ploughing can be calculated as

(6) $$\mu_{p}=\frac{A_{p}}{A_{s}}=\frac{R^{2}\cos^{-1}(1-\frac{h_{\mathrm{Pd}}}{R})-(R-h_{\mathrm{Pd}})\sqrt{2Rh_{\mathrm{Pd}}-h_{\mathrm{Pd}}{}^{2}}}{\pi (Rh_{\mathrm{Pd}}-\frac{1}{2}h_{\mathrm{Pd}}{}^{2})}$$

where *R* is the indenter radius, *h_Pd_* is the penetration depth, and *r* is the radius of the vertical projection semicircle. Equation (6) shows that the COF increased with *h_Pd_*, which explains why COF and *h_pd_* shared similar patterns of change and indicates that the COF is positively correlated with normal force, but negatively correlated with hardness.

The COF of adhesion can be calculated as

(7) $$\mu_{a}=\frac{k\tau_{s}A_{s}}{H_{s}A_{s}}=\frac{k\tau_{s}}{H_{s}}$$

where *k* is the ratio of adhesive area to contact area and *τ_s_* is the shear strength. According to Equation (7), the adhesion component of COF is almost constant. The change of COF mainly was mainly caused by the variation of the plough component of COF. As shown in Equation (6), a sudden change in hardness on both sides of the interface leads to a rapid change in the COF and *h_pd_* during the micro-friction tests. A low scratching normal pressure and a relatively high hardness usually indicate a deeper *h_Pd_* and higher COF; therefore, the values of *h_Pd_* and COF increase with normal pressure and present a rapid increase when sliding from hard friction components to the soft matrix. Nevertheless, because of the obvious deformation of the Fe/Cu interface along the sliding direction owing to its stronger bonding strength and better ductility, the changing trend of hardness at the interface slowed, causing a reduction in the rate of growth of *h_pd_* and COF in the interface area. The phenomenon that COF showed a decrease prior to an increase in the Cr/Cu interface under a normal force of 0.5 N was caused by the debonding of the Cr/Cu interface, which meant that Cr/Cu interface was no longer able to provide sufficient plough resistance during scratching. The micro-friction test results confirmed that the Fe/Cu interface has a higher strength than that of the Cr/Cu interface.

### 3.4. Macro-Friction Tests

#### 3.4.1. Wear Mechanism

The friction and wear performance of Cu–BCs containing Fe and Cr (samples 3# and 4#) was measured by a MM-3000 friction tester. Wear debris, subsurface (cross-section), and the worn surface were analysed after the macro-friction tests.

**Figure 12:**
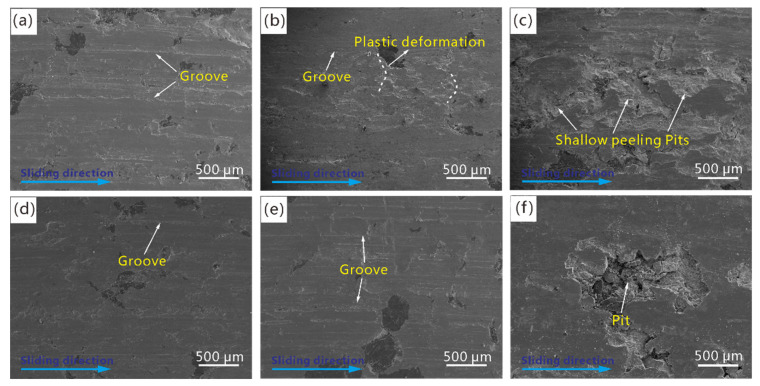
The worn surfaces of Cu–BCs containing Fe and Cr tested under different BEPUAs: (**a**) Fe low-BEPUA; (**b**) Fe medium-BEPUA; (**c**) Fe high-BEPUA; (**d**) Cr low-BEPUA; (**e**) Cr medium-BEPUA; and (**f**) Cr high-BEPUA.

The evolution of the worn surfaces of Cu–BCs under different BEPUAs is shown in Figure 12. The worn surface morphology of the Cu–BCs changed from a flat surface to a rugged and scarred surface with an increase in BEPUA.

Under a low BEPUA, regardless of the types of friction components contained in the Cu–BCs, the grooves parallel to the sliding direction were the main wear morphology. It is noted that the number and width of grooves on the worn surface of Cu–BC containing Fe is higher than that of Cu–BC with Cr. As the BEPUAs increased, the wear morphology of tested samples started to form obvious differences. For Cu–BC containing Cr, the density of grooves on the worn surface showed an increase tendency with BEPUA. However, for Cu–BC containing Fe, a wave-like wear morphology began to generate on the worn surface, which indicated strong local plastic deformation after the braking process, as shown in Figure 12b. By continuing to increase the BEPUA to a high level, the difference in the worn surface continued to increase for different tested samples. The worn surface of Cu–BC with Fe featured shallow pits with a high area ratio and a small number of grooves, as shown in Figure 12c. In addition, some undischarged fine particles can be found inside these pits. Nevertheless, the worn surface of Cu–BC with Cr exhibited lots of deep pits, and bits of friction components are exposed at the bottom of these pits, as shown in Figure 12f.

**Figure 13:**
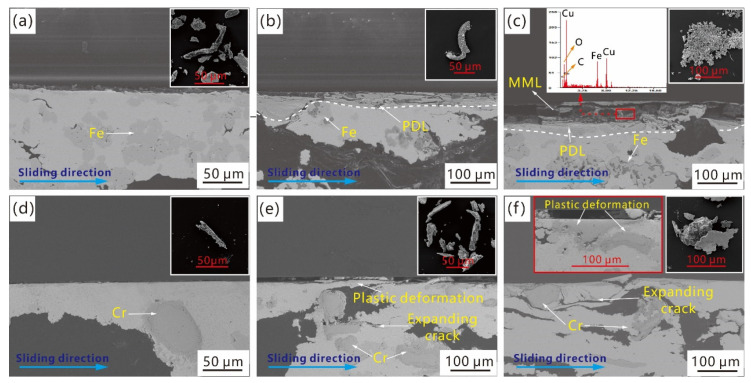
Subsurface and wear debris of Cu–BCs containing Fe and Cr tested under different BEPUA conditions: (**a**) Fe low-BEPUA; (**b**) Fe medium-BEPUA; (**c**) Fe high-BEPUA; (**d**) Cr low-BEPUA; (**e**) Cr medium-BEPUA; (**f**) Cr high-BEPUA.

Figure 13 shows the typical subsurface structures and wear debris of Cu–BCs containing different friction components under different BEPUAs. Under low BEPUA conditions, there was no obvious subsurface structure formed on the worn surfaces of the Cu–BCs containing Fe or Cr, as shown in Figure 13a,b. A continuous plastic deformation layer (PDL) with a thickness of 10–20 μm occurred on the worn surfaces of the Cu–BCs containing Fe, with an increase of BEPUA. However, limited plastic deformation of the Cu–BCs containing Cr led to the formation of thin incomplete PDLs, as shown in Figure 13e.

When the BEPUA increased to a higher level, the subsurface evolution pattern showed a more diverse changing trend for two kinds of Cu–BCs. For instance, as shown in Figure 13c, the Cu–BCs containing Fe showed a distinct three-layer structure, which consisted of a mechanical mixed layer (MML) formed by the accumulation of small particles of wear debris and second sintering under the influence of friction heat, as well as Fe-rich PDL and an undeformed matrix from top to bottom. However, the Cu–BCs containing Cr still did not form a typical subsurface structure, except for the generation of cracks beneath the surface of materials, as indicated in Figure 13f.

Under low and medium BEPUAs, the stripe-like shape was the main characteristic of wear debris among the Cu–BCs. The shape of the wear debris transformed from stripe-like to thin flake-like shapes on Cu–BCs containing Fe and to thick flake-like shapes for Cu–BCs containing Cr under high BEPUAs, as shown in Figure 13.

The mechanical properties of the friction components and interface bonding strength between the friction components and matrix were attributed to the difference in the evolutionary patterns of worn surfaces or subsurfaces between two kinds of Cu–BCs. Under low and medium BEPUAs conditions, owing to a better particle strengthening effect of Cr with higher hardness, Cu-BCs containing Cr showed a high resistance to plastic deformation. Therefore, plastic deformation had more difficulty developing on the worn surfaces of the Cu–BCs containing Cr compared with that of Cu–BCs containing Fe. The result, depth, and quantity of grooves on worn surface of Cu-BCs containing Cr were smaller than the other. In this condition, the plough was the main wear mechanism for Cu–BCs regardless of what friction component composites contained.

**Figure 14:**
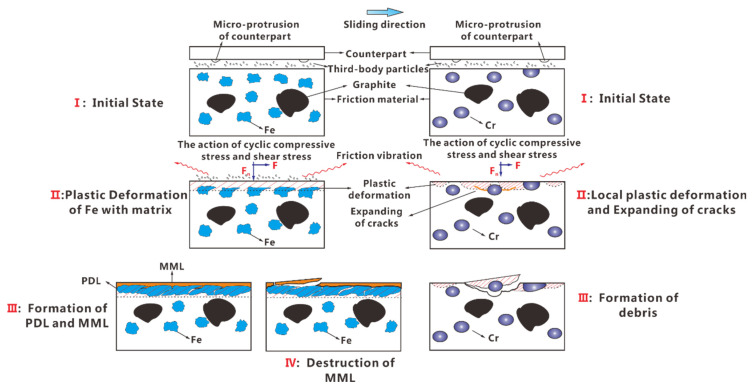
Evolution of the wear mechanism for Cu–BCs containing Fe and Cr under high BEPUA.

Under high BEPUA conditions, because of the poor wear resistance of the Cu matrix, the quality loss of the Cu matrix was much larger than that of Fe. With the surface of the Cu matrix being worn out, the exposed Fe phases started to spread along the sliding direction under repeated friction shear stress, leading to the formation of an Fe-rich PDL with high strength. Furthermore, under the premise of the stable existence of PDL, as the high-energy braking continued, under periodic high-frequency shear stress and compressive stress, the fine wear debris that remained on the worn surface tended to accumulate and connect to each other due to second sintering caused by friction heat, leading to the occurrence of MML. The wear of Cu–BCs containing Fe was caused by the peeling of MML due to the expansion of cracks along the rubbing direction, which were nucleated in the bonding defects and oxidation defects of MML. Therefore, the change in the wear mechanism from ploughing to MML destruction induced delamination, as shown on the left part in Figure 14.

Thanks to the effective particle strengthening of Cr, even under high BEPUA, the plastic deformation of the worn surface was not obvious for Cu–BCs containing Cr. However, the defect interface layer on Cr/Cu interface easily developed into the sources of cracks, causing the nucleation and propagation of cracks. As shown on the right part in Figure 14, when the cracks propagated to the surface of materials along the rubbing direction, the peeling of the copper matrix led to the wear of Cu–BCs containing Cr, resulting in the formation of thick flake-like wear debris. The wear mechanism transformed from ploughing- to matrix-peeling-induced delamination, as shown on the right side of Figure 13.

#### 3.4.2. Friction and Wear Behaviour

**Figure 15:**
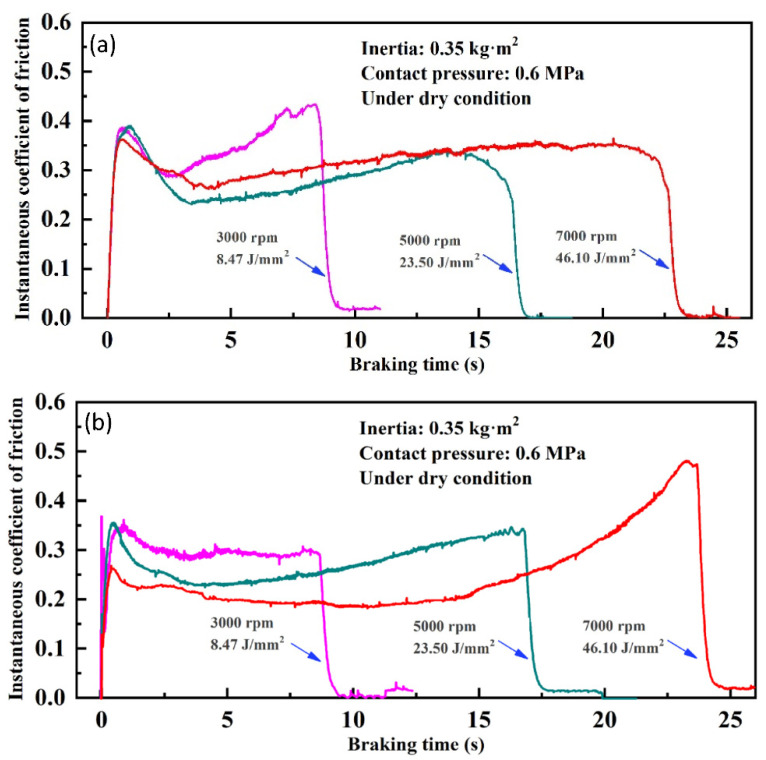
The instantaneous coefficients of friction of Cu–BCs containing Fe (**a**) and Cr (**b**).

The time-dependence curves of instantaneous COF under different BEPUAs are displayed in Figure 15. Typical saddle-like braking curves were visibly formed under nearly all test conditions. Furthermore, with an increase in BEPUA, the whole braking time showed a growth trend, but the COF displayed the opposite change tendency. Furthermore, Cu–BCs containing Fe showed more stable COF curves than those containing Cr under high BEPUA.

The formation of saddle-like braking curves is attributed to the change in friction surface morphology between the friction material and its counterpart during braking. The sudden braking shock was the main reason for the high COF in the early stages of braking. Then, with the generation of soft debris from the copper matrix or graphite at the friction interface, the instantaneous COF began to gradually decrease. During the end stage of braking, owing to a reduction in the amount of soft debris and the change from dynamic friction to static friction, the instantaneous COF increased again, eventually giving rise to the up-tail phenomenon of COF curves.

The differences in the braking performance of Cu–BCs containing different friction components under a high BEPUA resulted from the friction component characteristics and subsurface structural diversity. Fe’s contribution to the formation of oxidized MML on the worn surface, which featured relatively high hardness and strong heat resistance, helped to smooth the braking process. However, no obvious subsurface structure was formed on the worn surfaces of Cu–BCs containing Cr. The distinct decrease of COF in the middle stage of braking was mainly caused by a softening of the copper matrix due to friction heat accumulation. This explains why the COF of Cu–BCs containing Fe showed better stability.

**Figure 16:**
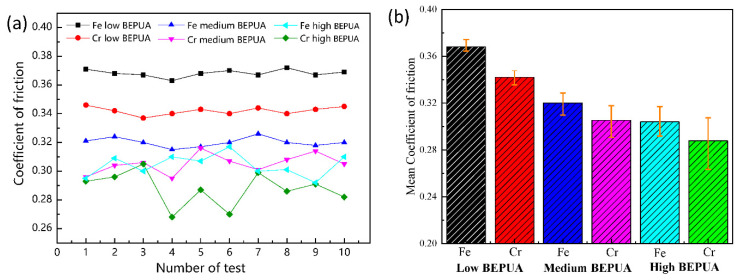
Variations in the coefficient of friction with the number of tests (**a**) and the mean coefficient of friction (**b**) of Cu–BCs containing Fe and Cr.

Figure 16 illustrates the variations in the mean COF over 10 cycles of braking for Cu–BCs containing Fe and Cr under different BEPUAs. The overall trend of COF showed a decrease with an increase in BEPUA for Cu–BCs containing two kinds of friction components. Between them, Cu–BCs containing Fe always showed a higher COF compared with those containing Cr under nearly all test conditions. In addition, under a high BEPUA, the fluctuation of COF in the Cu–BCs containing Fe was also relatively smaller than that of the Cu–BCs containing Cr, as shown in Figure 16a.

Well strength of diffusion bonding is beneficial to the function developing of Fe and Cr. Before the friction layer formed, the friction performance mainly depended on the intrinsic friction properties of each elements and their distribution area ratios. Under a low BEPUA, The absorption of braking energy by the deformation of worn surface was the main source of friction resistance. When the materials contain multiple components, assume that the normal pressure acting on each component is uniform, and the COF can be expressed as [28]

(8) $$\mu =\sum_{i=1}^{N}\left(\mu_{ip}+\mu_{ia}\right)\cdot \alpha_{i}$$

where *α_i_* is the covering area of each phase, *μ_ip_* is the ploughing COF of single component, and *μ_ia_* is the adhesion COF of single component.

Because Fe and Cr had the same volume content, the difference in friction performance was related to the frictional properties of Fe and Cr itself. According to the results of the micro-friction tests, *h_pd_* on the Fe particles was deeper under the same pressure, which resulted in intensive plastic deformation. This result indicates that the *μ_ip_* of Fe was much higher than that of Cr. Moreover, the adhesion tendency of Fe to alloy counterpart is much higher than that of Cr, which caused that the *μ_ia_* of Fe was higher than that of Cr as well. Hence, Cu-BCs containing Fe showed a higher COF, as shown in Figure 16.

With an increase in BEPUA, the surface temperature of the materials gradually raised, and the vibration intensified. Under these circumstances, the matrix softened, and more braking energy transformed into impact energy, leading to a decrease in the COF. Therefore, under a medium BEPUA, the COFs in the two kinds of Cu–BCs decreased observably. With a further increase in BEPUAs, the formed friction layer replaced the original friction surface, the tribology behavior of material is controlled by the structure and properties of friction layer. According to the fixed adhesion theory [29],

(9) $$\mu =\frac{\tau_{f}}{\sigma_{s}}$$

where *τ_f_* is the shear strength of the friction layer. Because the oxidized MML shows better mechanical properties than the exposed matrix, the composites containing Fe had a higher COF than that of the composites containing Cr. The vibration in the COF of Cu–BCs with Cr was related to the worn mechanism under a high BEPUA. The intense variations could be attributed to the participation of large size hard wear debris in braking process as a third body, due to peeling of Cr with flake-like wear debris.

**Figure 17:**
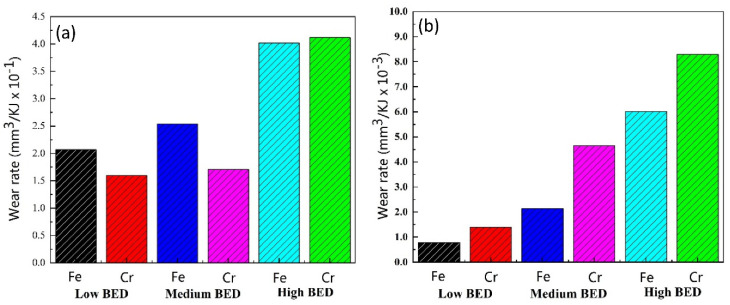
The wear rate of Cu–BCs and its counterpart: (**a**) wear rate of Cu–BCs and (**b**) wear rate of counterpart.

Figure 17 shows the wear rates of the Cu–BCs containing Fe and Cr under different BEPUAs. The wear rate increased with an increase of BEPUA. Under low and medium BEPUA values, the Cu–BCs containing Fe exhibited a higher wear rate than those containing Cr. Moreover, the wear rate increased rapidly for Cu–BCs containing Cr with BEPUA. However, when BEPUA reached a high level, a sharp increase was observed in the wear rates of the Cu–BCs with Cr, which began to exceed the wear rates of Cu–BCs with Fe. The wear rates of the counterparts followed a similar change pattern. Notably, the counterparts of Cu–BCs with Cr always showed a higher wear rate, as presented in Figure 17b.

The friction components were not destroyed under low and medium BEPUAs. In this situation, the friction components with the highest hardness were able to significantly improve the wear resistance of Cu–BCs. Consequently, Cu–BCs with Cr showed a lower wear rate under low and medium BEPUAs. However, under a high BEPUA, wear resistance was dependent on the subsurface structure. The Fe/Cu interface promoted the formation of MML with high hardness, thereby controlling the excessive wear of the Cu–BCs. No protective friction layer was formed on the worn surfaces of the Cu–BCs containing Cr under a high BEPUA, resulting in severe delamination wear.

The wear rates of the counterparts mainly related to the hardness of the friction components and the wear mechanism of the friction system. Under all tested conditions, Cr always showed stronger destruction capabilities than the tested counterparts. Therefore, the wear rates of the counterparts for the Cu–BCs containing Cr were higher, especially under a high BEPUA. The wear of the counterparts was further aggravated by delamination and the increase in tertiary bodies.

## 4. Conclusions

The following conclusions of this research can be drawn:

(1) A diffusion bonding interface was formed between Fe and the copper matrix, as well as between Cr and the copper matrix. Owing to the formation of the defect bonding layer, the Cr/Cu interface exhibited a lower bonding strength compared with that of the Fe/Cu interface. Therefore, Fe presents a better particle strengthening than Cr, which enhanced the strength of Cu–BCs more effectively.
(2) The COF of the Fe/Cu interface showed a slow growth trend owing to interface deformation. However, the COF of the Cr/Cu interface first indicated a decrease followed by a sharp increase in the interface area owing to local interface debonding, especially at a pressure of 0.5 N. Compared with Cr, Fe can reduced the rapidly variation of the COF on interface bonding area during the micro friction test.
(3) Fe is conducive to the improvement of COF, the Cu–BCs containing Fe showed higher COF values than that of Cu–BCs with Cr under all test conditions owing to its higher COF component. Cr is beneficial to an improvement of wear resistance because of its higher hardness, The Cu–BCs containing Cr presented a lower wear rate than that of Cu–BCs containing Fe.
(4) Under low and medium BEPUAs, ploughing was the main wear mechanism for all tested composites. However, under high BEPUAs, Fe promoted the formation of MML, meaning that the delamination induced by destruction of MML became the main wear mechanism. However, the addition of Cr increased the quantity of interface defects in Cu–BCs, promoting the nucleation and propagation of cracks in composites during braking, meaning that the delamination induced by peeling of matrix near friction surface became the main wear mechanism.

## Figures and Tables

**Table 1:** Chemical compositions of Cu–BCs (vol. %).

	Cu	Fe	Cr	Graphite
1#	90	10	\	\
2#	90	\	10	\
3#	50	10	\	40
4#	50	\	10	40

**Table 2:** Characteristics of the raw powders.

Raw Powders	Element Content	Particle Size
Cu	Cu ≥ 99.98 wt.%	<74 μm
Fe	Fe ≥ 99.8 wt.%	<74 μm
Cr	Cr ≥ 99.8 wt.%	<74 μm
Graphite	C ≥ 97.0 wt.%	150–600 μm

**Table 3:** Relative density and density of Cu–BCs.

No.	Sample	*ρ* *_th_*	*ρ* (g cm^−3^)	*ρ_r_*
1#	Cu–Fe	8.55	8.12	0.918
2#	Cu–Cr	8.14	7.42	0.912
3#	Cu–Fe–C	6.17	4.96	0.804
4#	Cu–Cr–C	5.20	4.12	0.793

**Table 4:** Microfriction test parameters for different areas.

Test Area	Fe/Cr Phases and Their Interface with Matrix
Test parameters	Radius of indenter: 10 μm, load: 0.3 N/0.5 N Sliding distance: 100 μm, sliding speed: 200 μm/min Relative humidity: 50%

**Table 5:** The test parameters of macro-friction.

Brake Pressure/MPa	Inertia/kg·m^2^	Rotation Speed/rpm	Theoretical BEPUA/J/mm^2^
0.6	0.35	3000/5000/7000	8.47/23.50/46.10

**Table 6:** EDS point results for the Cu–Fe interface in Figure 4b area A (at.%).

Point Number	Cu	Fe
1	60.34	39.66
2	18.71	81.29
